# Periodontal destruction indicators across different disease durations in older patients with type 2 diabetes: a cross-sectional study of nonlinear distribution patterns and selection bias

**DOI:** 10.3389/froh.2026.1953777

**Published:** 2026-09-22

**Authors:** Jie Yang, Xijun Zhu, Beibei Liang, Juan Zhang

**Affiliations:** 1 Department of Stomatology, Geriatric Hospital of Nanjing Medical University/Jiangsu Province Official Hospital/Jiangsu Province Geriatric Hospital, Nanjing, China; 2 Department of Endocrinology, Geriatric Hospital of Nanjing Medical University/Jiangsu Province Official Hospital/Jiangsu Province Geriatric Hospital, Nanjing, China

**Keywords:** aged, cross-sectional studies, diabetes mellitus type 2, disease duration, nonlinear dynamics, periodontitis/classification, selection bias

## Abstract

**Background/objectives:**

To investigate the distribution of periodontal destruction indicators across different disease duration stages in older patients with type 2 diabetes mellitus (T2DM), and to evaluate the nonlinear association between disease duration and periodontitis severity while addressing selection bias inherent in the cross-sectional design.

**Methods:**

A total of 70 T2DM patients aged 60–85 years were enrolled and divided into five groups according to disease duration: ≤5, 6–10, 11–15, 16–20, and >20 years. 100 age-matched non-diabetic individuals served as the reference group. Bleeding on probing (BOP), probing depth (PD), tooth loss due to periodontitis, and periodontitis stage, grade, and extent were recorded. Ordinal logistic regression models were established: (1) a baseline model adjusted for age and sex, and (2) a nonlinear model incorporating a quadratic term for disease duration.

**Results:**

The prevalence of periodontitis in the diabetic group was 97.1% (68/70), significantly higher than that in the reference group (45.0%, 45/100). The detection rate of teeth with PD >5 mm peaked in the 11–15-year group (87.5%) and declined to 70.0% in the >20-year group, whereas the mean number of tooth loss increased continuously with duration, reaching 6.2 teeth in the >20-year group. In the baseline model, all duration groups were associated with higher periodontitis stages, with odds ratios showing a non-linear pattern (peak OR = 18.89 in the 11–15 years group). In the nonlinear model, the quadratic term for duration was significant (*P* = 0.04), confirming an inverted U-shaped nonlinear relationship between disease duration and periodontitis stage.

**Conclusions:**

The detection rate of deep periodontal pockets and tooth loss exhibited a non-linear and dissociated distribution across disease duration groups in older T2DM patients, a pattern primarily driven by selection bias. The findings are descriptive, not causal. Prospective cohort studies incorporating comprehensive covariates, including glycemic control (HbA1c) and smoking status, are warranted to further validate the causal relationship.

## 1 Introduction

Periodontitis is a chronic inflammatory disease characterized by progressive destruction of the periodontal supporting tissues, with a high prevalence among middle-aged and older populations (1). Diabetes mellitus and periodontitis share a bidirectional relationship. Hyperglycemia can damage periodontal tissues through multiple mechanisms, while periodontal chronic inflammation may also adversely affect glycemic control (2–4).

The 2018 World Workshop on the Classification of Periodontal and Peri-Implant Diseases and Conditions introduced a new classification framework that incorporates the cumulative extent of periodontal destruction into staging and the risk of progression into grading, providing a more refined approach for evaluating the impact of diabetes on periodontal health (5–7). This classification framework integrates the cumulative extent of periodontal destruction (attachment loss, bone loss, tooth loss, and treatment complexity) into an ordered categorical variable, offering an ideal dependent variable for quantifying the cumulative effects of diabetes duration. However, most previous studies have treated diabetes as a binary variable (presence or absence) and have paid less attention to the distribution of periodontal destruction across different disease duration stages.

Notably, the relationship between diabetes duration and periodontal destruction may not follow a simple linear dose-response pattern. Although long-term hyperglycemia persistently promotes periodontal tissue damage, in late-stage disease, extensive tooth loss restructures the residual dentition and the observable indicators of periodontal pocketing, creating the illusion of a declining trend in deep pocket indicators among long-duration patients. This nonlinear evolution may be driven by competing risks (the natural decline in deep pocket detection rates in the residual dentition following the loss of severely affected teeth),duration-related treatment history (a higher probability of periodontal therapy in long-duration patients), and changes in host response (long-term high-glucose exposure may induce pathological tissue tolerance). These factors collectively require stratified analysis and nonlinear modeling to be properly elucidated. Therefore, treating diabetes merely as a binary variable would obscure this nonlinear dynamic evolution and the selection bias therein.

It should be noted that our research group has previously published a study on periodontal health in older diabetic patients (Yang J, et al. *Practical Geriatrics*, 2023); however, that study did not perform detailed duration stratification. The present study, based on endocrine-stomatology joint follow-up data from a geriatric hospital in Nanjing, represents the first duration-stratified analysis in older T2DM patients in this region. This population is characterized by complete long-term endocrinology follow-up records and reliable duration information, providing a unique clinical data foundation for exploring the nonlinear association between duration and periodontitis. The incremental contributions of this study are threefold: (1) it is the first to systematically describe the distribution of periodontitis stage, grade, and extent across different duration strata in older diabetic patients in this region; (2) it is the first to quantify the nonlinear dose-response pattern between duration and staging through the introduction of a quadratic term for statistical testing; and (3) it explicitly delineates the direction and magnitude of distortion caused by selection bias on the duration-periodontal destruction association in cross-sectional data, providing baseline data for the design of future prospective studies.

Based on the above literature gaps and theoretical background, we propose the following core hypotheses:

**Hypothesis 1:** Disease duration in older T2DM patients is **nonlinearly associated** with periodontitis stage—the detection rate of deep periodontal pockets peaks at mid-duration (approximately 11–15 years) and then declines with further prolongation of duration (i.e., an “inverted U-shaped” trend), whereas tooth loss continues to rise, resulting in a dissociated distribution.
**Hypothesis 2:** This “inverted U-shaped” distribution pattern is primarily explained by **selective retention bias** (changes in residual dentition composition following the loss of severely affected teeth in late-stage disease), rather than a true reversal of the biological effects of disease duration.

This study aims to test the above hypotheses through cross-sectional investigation and statistical modeling, and to discuss their implications for clinical interpretation.

## 2 Materials and methods

### 2.1 Study design and participants

This was a single-center cross-sectional analysis. A total of 70 patients with T2DM aged 60–85 years who underwent long-term follow-up at the Department of Endocrinology, Affiliated Geriatric Hospital of Nanjing Medical University, between October 2022 and December 2025, were enrolled. One hundred age-matched healthy individuals who undergoing health checkups during the same period were included as the non-diabetic reference group.

The inclusion criteria for the diabetic group were: (1) diagnosis of T2DM according to the 1999 WHO criteria; (2) disease duration ≥1 year; (3) age 60–85 years; and (4) ability to complete a full-mouth periodontal examination. Exclusion criteria were: (1) concomitant other immune-mediated or metabolic diseases; (2) receipt of periodontal treatment within the preceding 3 months; (3) severe cardiac, hepatic, or renal insufficiency; and (4) inability to cooperate with the examination due to cognitive impairment. Diabetic patients were divided into five groups according to disease duration verified from medical records: ≤5, 6–10, 11–15, 16–20, and >20 years.

The inclusion criteria for the reference group were: (1) no history of diabetes, with fasting plasma glucose and HbA1c within normal ranges; (2) no other severe systemic diseases; and (3) age 60–85 years.

This was an exploratory analysis for which no *a priori* sample size calculation was performed. To assess the support provided by the current sample size for the main findings, a *post-hoc* power analysis was conducted for the smallest statistically significant effect in the ordinal logistic regression (OR = 4.12), with *α* = 0.05 (two-sided). The statistical power (1-*β*) was 0.72, indicating that the current sample size is acceptable for detecting large effect sizes but insufficient for detecting moderate-to-small effects. In particular, the 6–10 years and >20 years groups each contained only 10 cases, limiting the statistical stability of estimates within these groups. This limitation should be fully considered in result interpretation and discussion, and the conclusions of this study should be regarded as exploratory findings rather than confirmatory conclusions.

### 2.2 Periodontal examination and examiner calibration

Periodontal examinations were performed according to the *Basic Periodontal Examination and Assessment Guidelines* (T/CHSA 002-2020) of the Chinese Stomatological Association. All examinations were conducted by a single trained dentist (Juan Zhang, attending physician, with 10 years of clinical and research experience in periodontology) using a Sogan SOGA-TI electronic probe. Calibration was performed before each examination session using a standard force of approximately 15 g.

Prior to the formal examination, the examining dentist and a deputy chief physician from the Department of Stomatology, Affiliated Geriatric Hospital of Nanjing Medical University (with 25 years of clinical experience, not involved in group assignment for this study) jointly performed full-mouth periodontal examinations on 10 older diabetic patients who did not meet the inclusion criteria, with re-examination after 72 hours. Agreement for Probing depth (PD) measurements was assessed using the intraclass correlation coefficient (ICC = 0.91, 95% CI: 0.85–0.95), and agreement for bleeding on probing (BOP) readings was assessed using Cohen's kappa (*κ* = 0.88), indicating excellent intra-examiner and examiner-gold standard agreement. During the formal examination, one randomly selected participant was re-examined after every 20 participants for quality control; the PD-ICC for repeated examinations ranged from 0.88 to 0.94.

PD and BOP were recorded at six sites per tooth (mesio-buccal, mid-buccal, disto-buccal, mesio-lingual, mid-lingual, and disto-lingual). The reasons for tooth loss were documented, and alveolar bone loss was assessed using oral radiographs. Only tooth loss definitively attributed to periodontitis was included in the tooth loss analysis; teeth lost due to trauma, caries, or other causes were excluded.

Smoking status was assessed using a structured questionnaire with three categories: “never smokers” (<100 cigarettes in a lifetime and currently not smoking), “former smokers” (≥100 cigarettes in a lifetime but quit ≥6 months ago), and “current smokers” (≥100 cigarettes in a lifetime and currently smoking, including daily and occasional smoking). Due to limitations of retrospective data collection, smoking status data were not completely available for all participants (missing rate: 2.9%, 2/70) and were therefore not included in the multivariate regression models. Smoking data were used only as an auxiliary criterion for grade C classification in periodontitis grading.

### 2.3 HbA1c measurement

Glycated hemoglobin (HbA1c) was measured by high-performance liquid chromatography (HPLC) using a Bio-Rad D-100™ Hemoglobin Testing System, with a normal reference range of 4.0%–6.0%. All blood samples were collected in the morning after 8–12 hours of fasting and were tested at the Central Laboratory of the Affiliated Geriatric Hospital of Nanjing Medical University. Due to limitations of retrospective data collection, HbA1c data were not completely available for all participants (missing rate: 5.7%, 4/70) and were therefore not included in the multivariate regression models. HbA1c was used primarily as an auxiliary criterion for grade C classification in periodontitis grading and for descriptive comparisons of glycemic control across duration groups (Table 2).

### 2.4 Diagnostic criteria

Diabetes was diagnosed according to the 1999 WHO criteria. Periodontitis diagnosis, staging, grading, and extent were determined according to the 2018 international classification (5–7). (1) **Staging**: Stages I–IV were assigned based on the severity of attachment loss, radiographic bone loss, number of teeth lost due to periodontitis, and treatment complexity (Table 1). (2) **Grading**: Grades A–C were assigned based on the rate of disease progression (bone loss/age ratio, smoking status, and glycemic control level). (3) **Extent**: Periodontitis was classified as localized when <30% of teeth were affected and as generalized when ≥30% of teeth were affected.

**Table 1:** Periodontitis staging criteria (2018 classification).

Stage	Severity	Complexity	Tooth loss due to periodontitis
I	CAL 1–2 mm	Max PD ≤4 mm, mainly horizontal bone loss	None
II	CAL 3–4 mm	Max PD ≤5 mm, mainly horizontal bone loss	None
III	CAL ≥5 mm	PD ≥6 mm, vertical bone loss ≥3 mm, etc.	≤4 teeth
IV	CAL ≥5 mm	Stage III complexity plus occlusal disturbance, <20 remaining teeth, etc.	≥5 teeth

Staging requires comprehensive case-by-case evaluation according to the full 2018 classification algorithm; this table summarizes only the key criteria.

CAL, clinical attachment loss; PD, probing depth.

### 2.5 Statistical analysis

Statistical analyses were performed using SPSS version 26.0 (IBM Corp, Armonk, NY, USA) and R version 4.2.1 (R Foundation for Statistical Computing, Vienna, Austria). Continuous variables are presented as mean ± standard deviation (x¯ ± s) and were compared among groups using one-way analysis of variance (ANOVA). Categorical variables are presented as numbers (percentages) and were compared using the chi-square test. Ordinal logistic regression models were used to evaluate the association between disease duration groups and periodontal severity (coded as 0 = no periodontitis, 1–4 = stage I-IV) as the ordered dependent variable. **Model A (baseline model)** used duration groups (≤5, 6–10, 11–15, 16–20, >20 years) as the independent variable, adjusted for age and sex. **Model B (nonlinear model)** treated duration as a continuous variable and introduced a quadratic term (duration^2^) to capture nonlinear trends, adjusted for age and sex. The likelihood ratio test (LRT) was used to compare models containing the quadratic term versus those with only the linear term. The proportional odds assumption was verified using the parallel lines test. Model fit was evaluated using AIC, BIC, and Nagelkerke pseudo-*R*^2^. A two-sided *P* < 0.05 was considered statistically significant.

## 3 Results

### 3.1 Baseline characteristics

The age and sex distributions of the reference group and the five diabetic duration groups are presented in Table 2. There were no significant differences in age or sex distribution among the groups (*P* > 0.05), indicating comparability between groups.

**Table 2:** Age and sex distribution across groups.

Group	*N*	Age (years, mean ± SD)	Male, *n* (%)	HbA1c (%, x¯ ± s)	Smokers/ever-smokers, *n* (%)
Reference	100	65.4 ± 6.3	60 (60.0)	–	32 (32.0)
≤5 years	20	65.2 ± 7.1	12 (60.0)	8.7 ± 1.7 (*n* = 19)	8 (40.0)
6–10 years	10	66.8 ± 6.5	6 (60.0)	8.9 ± 1.8 (*n* = 9)	3 (30.0)
11–15 years	16	67.5 ± 8.9	11 (68.8)	9.3 ± 2.1 (*n* = 15)	6 (37.5)
16–20 years	14	67.1 ± 9.2	9 (64.3)	9.1 ± 1.9 (*n* = 14)	5 (35.7)
>20 years	10	68.0 ± 8.4	6 (60.0)	9.2 ± 2.0 (*n* = 9)	3 (30.0)
*F*/*χ*^2^ value		0.42	0.56	0.92	4.58
*P* value		0.83	0.99	0.46	0.33

HbA1c and smoking data are reported descriptively only due to partial missingness and were not included in the regression models. Reference group HbA1c values are screening values and were not statistically compared with the diabetic groups.

### 3.2 Distribution of periodontal parameters and tooth loss

The prevalence of BOP in the reference group was 48.0%. All diabetic duration groups had higher BOP prevalence than the reference group, with the highest prevalence in the 16–20-year group (92.9%). The difference among all groups was statistically significant (*χ*^2^ = 18.34, *P* = 0.002); however, pairwise comparisons among the five diabetic groups showed no significant differences (*P* > 0.05).

The detection rate of teeth with PD 3–5 mm was higher in all diabetic groups than in the reference group (41.0%), but the difference among the five diabetic groups was not statistically significant (*χ*^2^ = 7.89, *P* = 0.09). The mean number of teeth with PD 3–5 mm was highest in the 11–15-year group (6.9 teeth).

The detection rate of teeth with PD >5 mm was 12.0% in the reference group and was significantly elevated in all diabetic groups (*P* < 0.001). It peaked in the 11–15-year group (87.5%) and then declined with further prolonged duration (78.6% in the 16–20-year group and 70.0% in the >20-year group). The mean number of teeth with PD >5 mm followed the same pattern.

Both the proportion of individuals with periodontitis-related tooth loss and the mean number of teeth lost increased continuously with diabetes duration. In the reference group, the prevalence of periodontitis-related tooth loss was 37.0%, with a mean of 1.7 teeth lost per person. In the >20-year duration group, the prevalence reached 100%, with a mean of 6.2 teeth lost per person. Between-group differences were significant for both measures (tooth loss prevalence: *χ*^2^ = 45.16, *P* < 0.001; mean tooth loss: *F* = 9.45, *P* < 0.001).

To further visualize the divergent trends in PD > 5 mm detection rate and mean tooth loss across duration groups, we constructed a dual *Y*-axis line chart (Figure 1). As shown, the two curves diverge after the 11–15 years group: the PD > 5 mm detection rate peaked in the 11–15 years group (87.5%) and then declined (70.0% in the >20 years group), whereas mean tooth loss continued to rise (from 1.8 teeth in the ≤5 years group to 6.2 teeth in the >20 years group). This divergence visually corroborates the statistical findings in Table 3.

**Figure 1:**
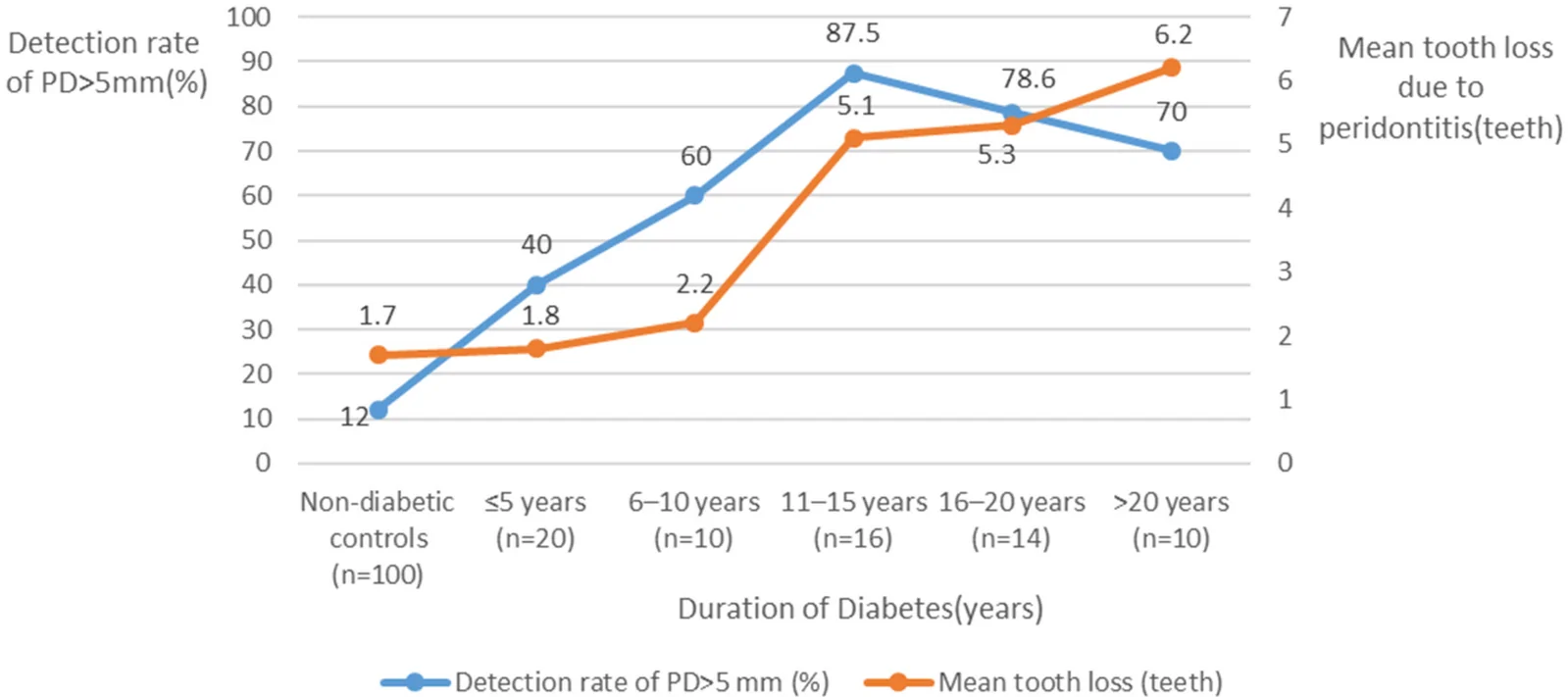
Dual *y*-axis line chart showing the detection rate of PD > 5 mm and mean tooth loss across diabetes duration groups.

**Table 3:** Periodontal parameters and tooth loss across groups.

Group	*N*	BOP+, *n* (%)	Mean BOP + sites	PD 3–5 mm (%)	Mean PD 3–5 mm (*n*)	PD >5 mm (%)	Mean PD >5 mm (*n*)	Tooth loss *n* (%)	Mean tooth loss (*n*)
Reference	100	48 (48.0)	5.6 ± 4.8	41.0	3.6 ± 3.8	12.0	0.3 ± 0.9	37 (37.0)	1.7 ± 2.7
≤5 years	20	14 (70.0)	6.2 ± 5.6	75.0	4.0 ± 3.9	40.0	1.3 ± 2.0	10 (50.0)	1.8 ± 2.2
6–10 years	10	8 (80.0)	7.8 ± 6.1	100.0	4.1 ± 3.3	60.0	1.7 ± 2.3	8 (80.0)	2.2 ± 2.4
11–15 years	16	14 (87.5)	8.9 ± 6.7	87.5	6.9 ± 6.0	87.5	4.1 ± 3.1	14 (87.5)	5.1 ± 4.5
16–20 years	14	13 (92.9)	10.2 ± 8.0	100.0	4.0 ± 2.9	78.6	2.4 ± 2.4	13 (92.9)	5.3 ± 3.4
>20 years	10	7 (70.0)	8.4 ± 7.9	70.0	3.3 ± 3.9	70.0	2.0 ± 1.9	10 (100.0)	6.2 ± 5.6
*χ*^2^/F		18.34	1.64	7.89	2.34	38.62	7.86	45.16	9.45
*P* value		0.002	0.15	0.09	0.05	<0.001	<0.001	<0.001	<0.001

### 3.3 Periodontitis stage, grade and extent

The prevalence of periodontitis was 45.0% (45/100) in the reference group and 97.1% (68/70) in the diabetic group (*χ*^2^ = 48.62, *P* < 0.001). The proportion of moderate-to-severe periodontitis (stages III + IV) increased with disease duration, reaching 100% in the >20-year group (trend *χ*^2^ = 11.26, *P* = 0.01) (Table 4). However, the distribution was not strictly monotonic across all groups. Percentages for stage, grade, and extent were calculated using the number of periodontitis patients in each group as the denominator to avoid confusion.

**Table 4:** Periodontitis stage, grade, and extent across groups [% (*n*/*N*)].

Group	Total *N*	Periodontitis patients	Stage I	Stage II	Stage III	Stage IV	Stage III/IV (%)	Grade A	Grade B	Grade C	Generalized
Reference	100	45	19.0% (19/100)	12.0% (12/100)	9.0% (9/100)	5.0% (5/100)	14.0%	51.1% (23/45)	35.6% (16/45)	13.3% (6/45)	20.0% (9/45)
≤5 years	20	18	44.4% (8/18)	11.1% (2/18)	22.2% (4/18)	22.2% (4/18)	44.4%	33.3% (6/18)	44.4% (8/18)	22.2% (4/18)	55.6% (10/18)
6–10 years	10	10	20.0% (2/10)	20.0% (2/10)	60.0% (6/10)	0% (0/10)	60.0%	20.0% (2/10)	60.0% (6/10)	20.0% (2/10)	20.0% (2/10)
11–15 years	16	16	0% (0/16)	12.5% (2/16)	62.5% (10/16)	25.0% (4/16)	87.5%	12.5% (2/16)	62.5% (10/16)	25.0% (4/16)	50.0% (8/16)
16–20 years	14	14	14.3% (2/14)	14.3% (2/14)	42.8% (6/14)	28.6% (4/14)	71.4%	28.6% (4/14)	28.6% (4/14)	42.9% (6/14)	57.1% (8/14)
>20 years	10	10	0% (0/10)	0% (0/10)	30.0% (3/10)	70.0% (7/10)	100.0%	0% (0/10)	50.0% (5/10)	50.0% (5/10)	100.0% (10/10)

Percentages for stage III/IV and generalized extent are calculated using the number of periodontitis patients in each group as the denominator.

Periodontitis grades did not differ significantly across the five diabetic groups (*χ*^2^ = 14.37, *P* = 0.26) (Table 4).

The proportion of generalized periodontitis generally increased with disease duration, with all 10 patients in the >20-year group having generalized disease (100%) (Table 4).

### 3.4 Ordinal logistic regression analysis

To evaluate the effect of duration grouping on periodontitis stage and to test for nonlinear trends, we performed ordinal logistic regression analyses (Table 5).

**Table 5:** Ordinal logistic regression analysis of disease duration and periodontitis severity.

Variable	Model A (baseline)	Model B (nonlinear)
Duration group (vs. Reference)		
≤5 years	4.12 (1.39–12.76)**	–
6–10 years	9.34 (1.90–52.91)**	–
11–15 years	18.89 (4.41–96.22)***	–
16–20 years	12.94 (3.29–60.08)***	–
>20 years	16.81 (3.58–93.78)***	–
Duration as continuous variable		
Linear term	–	0.089 (0.03–0.15)**
Quadratic term	–	−0.018 (−0.035 to −0.001)*
Covariates		
Age	1.05 (0.999–1.11)	1.05 (0.998–1.10)
Sex (male vs. female)	0.91 (0.49–1.69)	0.92 (0.50–1.71)
Model fit indices		
AIC	220.4	215.4
BIC	235.6	231.2
Nagelkerke *R*^2^	0.31	0.35
Parallel lines test *P*	0.12	0.14

Data are OR (95% CI); in Model B, the quadratic term for duration is presented as the regression coefficient (*β*). Model A: adjusted for age and sex, with duration groups as categorical variables; Model B: adjusted for age and sex, with duration as a continuous variable and quadratic term. In Model B, the linear term coefficient was positive (*β* = 0.089), while the quadratic term coefficient was negative (*β* = −0.018, *P* = 0.04), indicating a downward-opening parabola (inverted U-shape)—i.e., the stage first increased and then decreased with duration. The statistical significance of the quadratic term (*P* = 0.04) confirms the nonlinear trend; the inflection point was estimated at approximately 12–14 years of duration (calculated as −*β*₁/(2×*β*₂) = −0.089/(2×−0.018) ≈ 2.47 coding units), consistent with the highest OR observed in the 11–15 year group in Model A. The LRT showed that the nonlinear model outperformed the linear model (*χ*² = 4.28, *P* = 0.04). Both Model A and Model B were adjusted for age and sex; neither reached statistical significance (age OR = 1.05, *P* = 0.058; sex OR = 0.91, *P* = 0.77). The parallel lines test supported the proportional odds assumption (*P* > 0.05 for both).

AIC, Akaike information criterion; BIC, Bayesian information criterion.

* *P* < 0.05.

** *P* < 0.01.

*** *P* < 0.001.

**Model A (baseline model):** Adjusted for age and sex only. All duration groups were positively associated with higher periodontitis stages. The 11–15 years group had the highest point estimate of OR (OR = 18.89, 95% CI: 4.41–96.22); although the ORs for the subsequent groups (16–20 years, >20 years) decreased somewhat, they remained statistically significant, showing a non-monotonic pattern of first increasing and then decreasing. The age effect approached borderline significance (OR = 1.05, *P* = 0.058), while the sex effect was not significant (OR = 0.91, *P* = 0.771).

**Model B (nonlinear model):** Duration was treated as a continuous variable with a quadratic term, still adjusted for age and sex. The quadratic term coefficient was −0.018 (*P* = 0.04). The likelihood ratio test (LRT) showed that the model with the quadratic term had significantly better fit than the model with only the linear term (*χ*^2^ = 4.28, *P* = 0.04, AIC decreased from 218.6 to 215.4), indicating an **inverted U-shaped nonlinear** relationship between duration and periodontitis stage—i.e., stage first increased and then decreased with duration, with an inflection point at approximately 12–14 years.

**Model diagnostics:** The parallel lines test supported the proportional odds assumption (*χ*^2^ = 15.32, *P* = 0.12); VIF values for all independent variables ranged from 1.02 to 1.18, indicating no significant multicollinearity.

## 4 Discussion

### 4.1 Nonlinear dissociated distribution of deep pockets and tooth loss: selection bias as the primary explanation

This study found that in older T2DM patients, the detection rate of deep periodontal pockets (PD > 5 mm) and tooth loss exhibited a non-linear dissociated distribution across duration groups: the deep pocket detection rate was highest in the 11–15 years group (87.5%, mean 4.1 teeth per person) and then declined, whereas the number of teeth lost continued to increase with duration, reaching a mean of 6.2 teeth in the >20-year group. The nonlinear test in Model B further confirmed the statistical significance of this trend, supporting the existence of an inverted U-shaped nonlinear relationship.

How should this phenomenon be understood? We propose that the primary mechanism is “selective retention bias”—a common epidemiological bias in cross-sectional studies, also known as “depletion of susceptibles” (8). Specifically, in the later stages of disease, a substantial number of severely affected teeth have been extracted or exfoliated, and the denominator for assessing deep periodontal pockets—the remaining dentition—has fundamentally changed. The decline in deep pocket detection rate does not indicate improvement in periodontal health but rather reflects a statistical artifact caused by the exclusion of severely diseased teeth. This phenomenon is not uncommon in chronic disease epidemiology. For example, in survival analyses of patients with advanced heart failure, the cardiac function of survivors may appear better than that of non-survivors, but this does not imply “improvement of cardiac function with disease progression.” Additionally, patients with longer disease duration are more likely to have received prior periodontal treatment (including scaling, root planing, and extraction of non-salvageable teeth), which may further lower PD levels in the later-stage groups.

We do not claim that the above findings represent a new biological mechanism. Rather, we explicitly characterize them as a quantitative description of “how selection bias distorts the duration-periodontal destruction association” in cross-sectional data. Although this bias phenomenon is not itself a new discovery, it has rarely been systematically quantified through duration stratification in older diabetic populations, let alone statistically tested by introducing a quadratic term. The significance of this study lies in providing clinicians with a set of quantifiable reference data, enabling them to recognize, when facing long-duration diabetic patients, that a decrease in deep pockets in the remaining dentition does not equal improvement in periodontal health—behind it lies the filtering effect of tooth loss on the residual dentition.

Based on the above distributional characteristics, we propose the working hypothesis of “**accumulation–outbreak–tooth loss compensation**”: early stage (≤5 years) characterized primarily by gingival inflammation and mild pocketing (accumulation); middle stage (11–15 years) characterized by widespread deep pocketing and the most prominent periodontal destruction (outbreak); and late stage (>15 years) characterized by a decline in deep pocket detection rate due to extensive tooth loss (tooth loss compensation). The nonlinear trend in Model B is consistent with this hypothesis. This hypothesis is inferred from cross-sectional distributions and the definitive longitudinal trajectory requires prospective cohort validation.

### 4.2 Duration associated with stage, not with grade

In this study, diabetes duration showed a strong association with periodontitis stage (reflecting cumulative destruction) (all duration groups significant in Model A, quadratic term significant in Model B), but no significant association with grade (reflecting progression rate) (*χ*^2^ = 14.37, *P* = 0.26).

Stage reflects cumulative lifetime damage. With increasing diabetes duration, advanced glycation end-products continue to accumulate in periodontal tissues, microvascular pathology progressively worsens, and tissue repair capacity progressively declines, collectively increasing the total burden of periodontal destruction over time. Grade reflects current rate of damage. According to the 2018 classification, grade is primarily determined by the bone loss/age ratio, smoking status, and glycemic control level (HbA1c) (5–7). Among these, glycemic control and smoking behavior are modifiable factors. A patient with a 20-year history of diabetes who maintains HbA1c < 7.0% and is a non-smoker may be assigned grade B or even grade A, whereas a patient with only a 5-year history but with HbA1c > 9.0% and who smokes could be assigned grade C. Therefore, grade primarily depends on currently active modifying factors rather than the length of disease duration, consistent with our findings.

**Clinical implication:** when assessing periodontal risk in diabetic patients, duration and glycemic control provide different dimensions of information and are not interchangeable.

### 4.3 Comparison with prospective cohort studies (Genco, Preshaw, and others)

Our findings should be contextualized within the evidence base of prospective cohort studies. Genco and Borgnakke systematically summarized the epidemiological evidence on the diabetes-periodontitis association, clearly noting that longitudinal studies show diabetes frequently precedes periodontitis, suggesting a causal role, and that diabetic patients have a significantly elevated risk of tooth loss (2). Evidence from the Pima Indian longitudinal cohort indicates that diabetes is an independent risk factor for alveolar bone loss. Taylor et al. further reported that over a 2-year follow-up period, the risk of alveolar bone loss progression in T2DM patients was more than 4 times that of non-diabetic individuals, and that those with poor glycemic control (HbA1c > 9%) had a risk increased to 11-fold (9). Preshaw et al. further noted that diabetic patients have approximately a 3-fold increased susceptibility to periodontitis, with a dose-response relationship between hyperglycemia and periodontitis severity (3).

Compared with these classic studies, our study differs in the following analytical perspectives: (1) The above studies mostly treated diabetes as a binary variable or focused on the association between glycemic control level (HbA1c) and periodontitis, with less systematic exploration of the independent and nonlinear role of duration in periodontitis staging; (2) Genco et al.'s study on patients with type 1 diabetes of over 50 years’ duration suggested that very long-duration groups may have unique periodontal manifestation patterns, but this population differs from our older T2DM patients in terms of disease natural history and is not directly generalizable; (3) The dose-response relationship emphasized by Preshaw et al. more strongly reflects the “intensity” of glycemic control (HbA1c) rather than “duration,” suggesting that duration and glycemic control level may be two independent and interacting dimensions (3).

The incremental contribution of this study lies in: being the first to systematically quantify, in an older Chinese T2DM population, the cross-group distributional differences of duration-stratified periodontal destruction indicators using the 2018 classification framework of staging and grading; being the first to statistically verify the existence of an inverted U-shaped nonlinear trend through the introduction of a quadratic term for duration; and clearly delineating the directional distortion of selection bias on cross-sectional associations. This provides baseline data and effect size estimates for the future design of prospective cohort studies incorporating complete covariates including HbA1c, smoking, plaque index, and periodontal treatment history.

### 4.4 Clinical implications

Although the present study cannot establish causal relationships or definitive temporal trajectories, the distributional differences across duration groups offer some clinically suggestive insights: First, the ≤5-year group had few deep pockets and limited tooth loss, suggesting that this phase may represent a critical window for periodontal basic treatment and patient education; Second, the 11–15-year group exhibited the most prominent periodontal destruction indicators, warranting comprehensive periodontal evaluation every 3–6 months and active implementation of sequential periodontal therapy; Third, the >15-year group, despite a decline in deep pocket detection, showed continued increase in tooth loss, suggesting that the clinical focus should shift from pocket control to preserving the remaining dentition and functional rehabilitation.

### 4.5 Methodological limitations

Several methodological limitations should be acknowledged. First, the cross-sectional design precludes causal inference; the “rise then fall” pattern reflects between-group differences at a single time point, not within-individual changes over time. The nonlinear trend in Model B provides statistical evidence of shape, not directionality.

Second, the sample size was limited (70 patients), with subgroups as small as 10 cases, resulting in wide confidence intervals and insufficient power to detect moderate-to-small effects. We have presented all percentages in the “% (n/N)” format in Table 4 to enhance transparency for small subgroups.

Third, key covariates were missing. The regression models were adjusted only for age and sex, and could not include known determinants of periodontitis progression (e.g., HbA1c, smoking, plaque score, prior periodontal therapy) due to incomplete recording in this retrospective database, introducing potential unmeasured confounding. Thus, the ORs in Table 5 represent age- and sex-adjusted associations, not independent net effects of duration. Moreover, the periodontitis staging criteria inherently include tooth loss, creating definitional overlap between the dependent variable and late-stage disease. Together, these limitations suggest that our findings should be interpreted as a description of cross-sectional distributional characteristics rather than an unbiased estimate of the net effect of duration on periodontal health.

## 5 Conclusions

In this cross-sectional study, the detection rate of deep periodontal pockets and tooth loss in older T2DM patients showed a non-linear, dissociated pattern across disease duration—the deep pocket detection rate peaking at 11–15 years and then declining (*P* = 0.04 for quadratic term, confirming an inverted U-shaped trend), whereas tooth loss continued to rise. This pattern is best explained as statistical artifact due to selective retention bias, not as a true reversal of biological effects. Given incomplete covariate data (HbA1c, smoking), regression was adjusted for age and sex only; accordingly, the present findings should be interpreted as descriptive rather than causal. The proposed “accumulation–outbreak–tooth loss compensation” hypothesis and quantified non-linear association provide baseline estimates for future prospective cohort studies.

## Data Availability

The raw data supporting the conclusions of this article will be made available by the authors, without undue reservation.

## References

[1] Wang X. The Fourth National Oral Health Epidemiological Survey Report. Beijing, China: People’s Medical Publishing House (2018).

[2] Genco RJ Borgnakke WS. Diabetes as a potential risk for periodontitis: association studies. Periodontol 2000. (2020) 83:40–45. 10.1111/prd.12270 32385881

[3] Preshaw PM Alba AL Herrera D Jepsen S Konstantinidis A Makrilakis K. Periodontitis and diabetes: a two-way relationship. Diabetologi. (2012) 55:21–31. 10.1007/s00125-011-2342-y PMC3228943 22057194

[4] Wu CZ Yuan YH Liu HH Li SS Zhang BW Chen W. Epidemiologic relationship between periodontitis and type 2 diabetes mellitus. BMC Oral Health. (2020) 20:204. 10.1186/s12903-020-01180-w 32652980 PMC7353775

[5] Tonetti MS Greenwell H Kornman KS. Staging and grading of periodontitis: framework and proposal of a new classification and case definition. J Periodontol. (2018) 89(Suppl. 1):S159–S172. 10.1002/JPER.18-0006 29926952

[6] Papapanou PN Sanz M Buduneli N Dietrich T Feres M Fine DH. Periodontitis: consensus report of workgroup 2 of the 2017 world workshop on the classification of periodontal and peri-implant diseases and conditions. J Periodontol. (2018) 89(Suppl. 1):S173–S182. 10.1002/JPER.17-0721 29926951

[7] Jepsen S Caton JG Albandar JM Bissada NF Bouchard P Cortellini P. Periodontal manifestations of systemic diseases and developmental and acquired conditions: consensus report of workgroup 3 of the 2017 world workshop on the classification of periodontal and peri-implant diseases and conditions. J Periodontol. (2018) 89(Suppl. 1):S237–S248. 10.1002/JPER.17-0733 29926943

[8] Hernán MA Hernández-Díaz S Robins JM. A structural approach to selection bias. Epidemiology. (2004) 15(5):615–625. 10.1097/01.ede.0000135174.63482.43 15308962

[9] Taylor GW Burt BA Becker MP Genco RJ Shlossman M Knowler WC. Non-insulin-dependent diabetes mellitus and alveolar bone loss progression over 2 years. J Periodontol. (1998) 69(1):76–83. 10.1902/jop.1998.69.1.76 9527565

